# Comparative effects of calcium, vitamin D, and combined supplementation on growth and bone mineral density in healthy children and adolescents: a systematic review and network meta-analysis

**DOI:** 10.3389/fnut.2026.1979566

**Published:** 2026-10-16

**Authors:** Guochun Lv, Pan Liu, Jing He, Fei Qu, Yunya Zhou, Shiwei Wang, Hong Xiang, Hao Gou

**Affiliations:** 1 School of Clinical Medicine, Chengdu University of Traditional Chinese Medicine, Chengdu, China; 2 Hospital of Chengdu University of Traditional Chinese Medicine, Chengdu, China

**Keywords:** adolescents, bone mineral density, calcium, children, growth velocity, network meta-analysis, vitamin D

## Abstract

**Background:**

Calcium and vitamin D are central to skeletal development in childhood, yet the relative effects of calcium alone, vitamin D alone, and their combination on somatic growth and bone mineral density (BMD) in healthy children and adolescents remain unclear.

**Methods:**

Eight databases were searched from inception through June 2026 for randomized controlled trials of oral calcium and/or vitamin D supplementation. Random-effects pairwise meta-analyses and Bayesian network meta-analysis (NMA) evaluated monthly height growth, monthly weight gain, and BMD at the lumbar spine, femoral neck, and total body.

**Results:**

Sixteen RCTs involving approximately 2,100 participants were included. Supplementation did not significantly affect monthly height growth (MD = 0.0441 cm/month, 95% CI [−0.0114, 0.0996]) or monthly weight gain (MD = 0.0075 kg/month, 95% CI [−0.0064, 0.0214]). Small positive differences were observed for lumbar spine BMD (MD = 0.0145 g/cm^2^, 95% CI [0.0040, 0.0249]), femoral neck BMD (MD = 0.0101 g/cm^2^, 95% CI [0.0064, 0.0138]), and total body BMD (MD = 0.0110 g/cm^2^, 95% CI [0.0049, 0.0170]), with substantial heterogeneity for lumbar spine and total body outcomes. Age-subgroup findings were exploratory, and no significant subgroup differences were found by intervention duration. The NMA did not establish consistent superiority of any active regimen.

**Conclusion:**

Calcium and/or vitamin D supplementation did not appreciably accelerate height or weight gain. Small site-specific increases in aBMD were observed, but their clinical significance remains uncertain and they do not establish benefits for bone strength or fracture prevention. Supplementation should primarily address inadequate intake, biochemical deficiency, or other clinical indications rather than routine growth promotion.

**Systematic review registration:**

https://www.crd.york.ac.uk/prospero/, identifier CRD420261388414.

## 1 Introduction

Childhood and adolescence are critical periods for longitudinal growth and skeletal mineral accrual. Peak bone mass is typically achieved in early adulthood and strongly influences later susceptibility to osteoporosis and fractures (1). Approximately 40%–60% of adult bone mass is accrued during the pubertal growth spurt (2), making childhood and adolescence a key window for interventions that support skeletal health (3). Adequate nutrition is also essential for normal physical development, immune function, and overall growth (4).

Calcium is the principal inorganic component of the bone matrix. Vitamin D serves a complementary role by promoting intestinal absorption of calcium and phosphorus, regulating parathyroid hormone secretion and bone metabolism, and maintaining mineral homeostasis (5, 6). Inadequate calcium intake and low vitamin D status remain common worldwide. Low serum 25-hydroxyvitamin D [25(OH)D] concentrations are especially prevalent among children (7), and calcium intake is often insufficient in populations with limited dairy consumption and in many low and middle income settings (8). Severe insufficiency of either nutrient can compromise mineralization and increase the risk of nutritional rickets (9). Pediatric bone health depends on an adequacy rather than indiscriminate excess.

Recommended intakes vary across countries and regions because of differences in age classifications, sunlight exposure, dietary patterns, target populations, and guideline development methods (10, 11). Current recommendations generally set calcium requirements for children and adolescents at approximately 800–1,300 mg/d. Vitamin D recommendations are commonly about 400 IU/d during infancy and 400–800 IU/d from 1 to 18 years of age (10, 12, 13). Despite broad agreement that both nutrients are fundamental to pediatric bone health, their relative effects, when given alone or together on growth, development, and BMD remain unresolved. Direct head-to-head comparisons are scarce, and no preferred regimen has been established for maximizing growth velocity or bone mass accrual during sensitive developmental periods (14, 15).

We systematically reviewed randomized controlled trials and performed a Bayesian network meta-analysis comparing calcium, vitamin D, and combined calcium plus vitamin D supplementation in healthy children and adolescents. The objectives were to: (1) quantify effects on monthly height growth, monthly weight gain, and BMD at the lumbar spine, femoral neck, and total body; (2) compare the relative efficacy of the three supplementation strategies and establish probabilistic rankings; (3) investigate potential effect modifiers, including age and intervention duration; (4) assess the robustness of the findings through heterogeneity, consistency, sensitivity. This analysis was designed to provide comparative evidence for pediatric nutrition and bone health practice.

## 2 Methods

### 2.1 Protocol and registration

This study was prepared in accordance with the PRISMA-NMA guidelines for systematic reviews and network meta-analyses (16). Its protocol was registered in PROSPERO (CRD420261388414). Registration preceded study screening, data extraction, risk-of-bias assessment, and quantitative synthesis. Database searches had been completed before registration, and no additional searches were conducted afterward. Because the analysis used only previously published randomized controlled trial data, additional ethical approval and informed consent were not required.

### 2.2 Search strategy

From inception through June 2026, searches were performed in PubMed, Web of Science, Embase, the Cochrane Library, China National Knowledge Infrastructure (CNKI), Wanfang Data, the VIP Database, and the Chinese Biomedical Literature Database (SinoMed). The strategy combined Medical Subject Headings (MeSH) with free text keywords such as “vitamin D,” “calcium,” “supplements,” “children,” and “randomized controlled trial.” No language restriction was imposed at the search stage. Database-specific strategies are provided in Supplementary Material 1.

### 2.3 Criteria for inclusion/exclusion of studies

Eligibility was defined with the PICO framework. studies were included if they: (1) used a randomized controlled trial design to examine vitamin D and/or calcium supplementation for growth, development, or BMD; (2) enrolled children or adolescents who were otherwise healthy at study entry; (3) administered vitamin D and/or calcium orally; (4) included a placebo or non-supplemented control; (5) reported at least one eligible outcome: height, body weight, or BMD.

For this review, “healthy” was defined as the absence of a diagnosed chronic disease or disorder expected to materially affect linear growth, bone metabolism, or nutrient absorption. Isolated low calcium intake, low vitamin D status, or a growth concern was permitted when participants were otherwise healthy and no underlying growth, endocrine, gastrointestinal, renal, or metabolic bone disorder had been diagnosed. Studies were excluded when quantitative data were incomplete or unsuitable for analysis, full text was unavailable, the publication language was neither Chinese nor English, records duplicated another database entry, supplementation was administered by a nonoral route such as intramuscular injection, or the design was nonrandomized. Nonrandomized comparative studies, case reports, protocols, reviews, conference abstracts, and clinical guidelines were also ineligible.

### 2.4 Study screening and data extraction

Two reviewers independently screened the records and extracted data. After duplicates were removed in EndNote 20, titles and abstracts were evaluated, followed by full text assessment of potentially eligible reports against the prespecified criteria.

Data were entered into a standardized form covering the first author, publication year, country, sample size, participant age, sex distribution, intervention type and dose, and intervention duration. Disagreements were settled through discussion, with a third investigator consulted when necessary.

For height, body weight, and BMD, change-from-baseline values were used for quantitative synthesis. Directly reported change scores were extracted when available; otherwise, mean changes were calculated as the follow-up value minus the baseline value. For height and body weight, follow-up duration was additionally extracted to derive monthly growth rates. For BMD outcomes, the skeletal site, measurement technique, DXA system, calibration information, measurement scale, and body- or bone-size adjustment were reviewed against the original reports. Only anatomically corresponding DXA-derived areal BMD outcomes were pooled, with lumbar spine, femoral neck, and total body BMD analyzed separately and standardized to g/cm^2^. pQCT-derived volumetric BMD and non-equivalent skeletal regions were excluded from the corresponding pooled analyses. Residual methodological differences were considered potential sources of heterogeneity. Age- or sex-standardized growth z-scores and height-adjusted BMD were not consistently available and therefore could not be synthesized.

### 2.5 Risk-of-bias assessment

Risk of bias was assessed with the Cochrane Risk of Bias 2 (RoB 2) tool. Two reviewers independently evaluated the five domains specified in the Cochrane Handbook for Systematic Reviews of Interventions: the randomization process, deviations from intended interventions, missing outcome data, outcome measurement, and selection of the reported result. Disagreements were settled by consensus or adjudication by a third reviewer.

### 2.6 Statistical analysis

Conventional pairwise meta-analyses were conducted for each outcome using random-effects models, with pooled effects expressed as mean differences (MDs) and 95% confidence intervals (CIs) and between-study heterogeneity summarized using I^2^.

For height and body weight, monthly changes were calculated as $\left({\bar{X}}_{f\,i\,n\,a\,l}-{\bar{X}}_{b\,a\,s\,e\,l\,i\,n\,e}\right)$/t, where t denotes follow-up duration in months. When the standard deviation (SD) of change score was unavailable, it was estimated as SD_change_ = $\sqrt{{\text{SD}}_{\text{baseline}}^{2}+{\text{SD}}_{\text{final}}^{2}-2\,\text{r}\times {\text{SD}}_{\text{baseline}}\times {\text{SD}}_{\text{final}}}$, assuming *r* = 0.90. A sensitivity analysis assuming *r* = 0.80 yielded similar results. Monthly standardization was used only to account for differences in follow-up duration. Because age- or sex-standardized growth measures and height-adjusted BMD were not consistently available, pooled estimates were derived from randomized between-group contrasts within each trial using common outcome scales. Accordingly, these estimates were interpreted as study-level average treatment effects rather than individual effects standardized for age or body size. Exploratory subgroup analyses examined age (3–6, 6–11, and >11 years) and intervention duration (≤12 vs. >12 months), with studies (k) and participants (N) reported for each subgroup. Leave-one-out sensitivity analyses and meta-regression were performed where appropriate. In multi-arm trials, one active-arm comparison was included in pairwise analyses to avoid double-counting shared controls; the arm was selected according to the prespecified supplementation category and dose, independently of observed effects. In the network meta-analysis, all eligible arms of multi-arm trials were retained under the same study identifier and modeled simultaneously. The shared comparator arm was entered only once. The standard multi-arm trial parameterization implemented in gemtc accounts for the dependency among treatment effects arising from a common comparator, thereby preserving the within-study correlation structure and avoiding artificial inflation of the statistical weight of the shared control group.

Bayesian NMA was implemented in R version4.4.1 using the gemtc package, with four independent Markov chain Monte Carlo (MCMC) chains, 20,000 adaptation iterations, and 50,000 sampling iterations per chain. Convergence was assessed using trace and posterior density plots and the Brooks-Gelman-Rubin statistic. Network estimates were reported as MDs with 95% credible intervals (CrIs). SUCRA values summarized posterior rankings and were interpreted alongside effect sizes and uncertainty, not as evidence of superiority. Network inconsistency was assessed when permitted by the structure of the available evidence. Consistency and inconsistency models were compared. Local inconsistency was further assessed using node-splitting when direct and indirect evidence could be separately estimated. When data permitted, small-study effects were explored using comparison-adjusted funnel plots; symmetry was not considered evidence of the absence of publication bias.

## 3 Results

### 3.1 Study selection and included studies

Across the eight databases, 2,465 records were identified. After 794 duplicates were removed, 1,557 records were excluded during title and abstract screening, and the full texts of 11 records could not be obtained. Consequently, 103 full text reports were assessed for eligibility. Of these, 87 were excluded because they involved dairy products or soluble corn fiber (*n* = 28), incorporated exercise training programs (*n* = 8), used ineligible study designs (*n* = 26), enrolled children with diseases (*n* = 6), were reviews or meta-analyses (*n* = 6), did not report relevant outcomes (*n* = 10), or were duplicate or overlapping publications (*n* = 3). Ultimately, 16 randomized controlled trials (17–32) were included in the meta-analysis (Figure 1).

**FIGURE 1:**
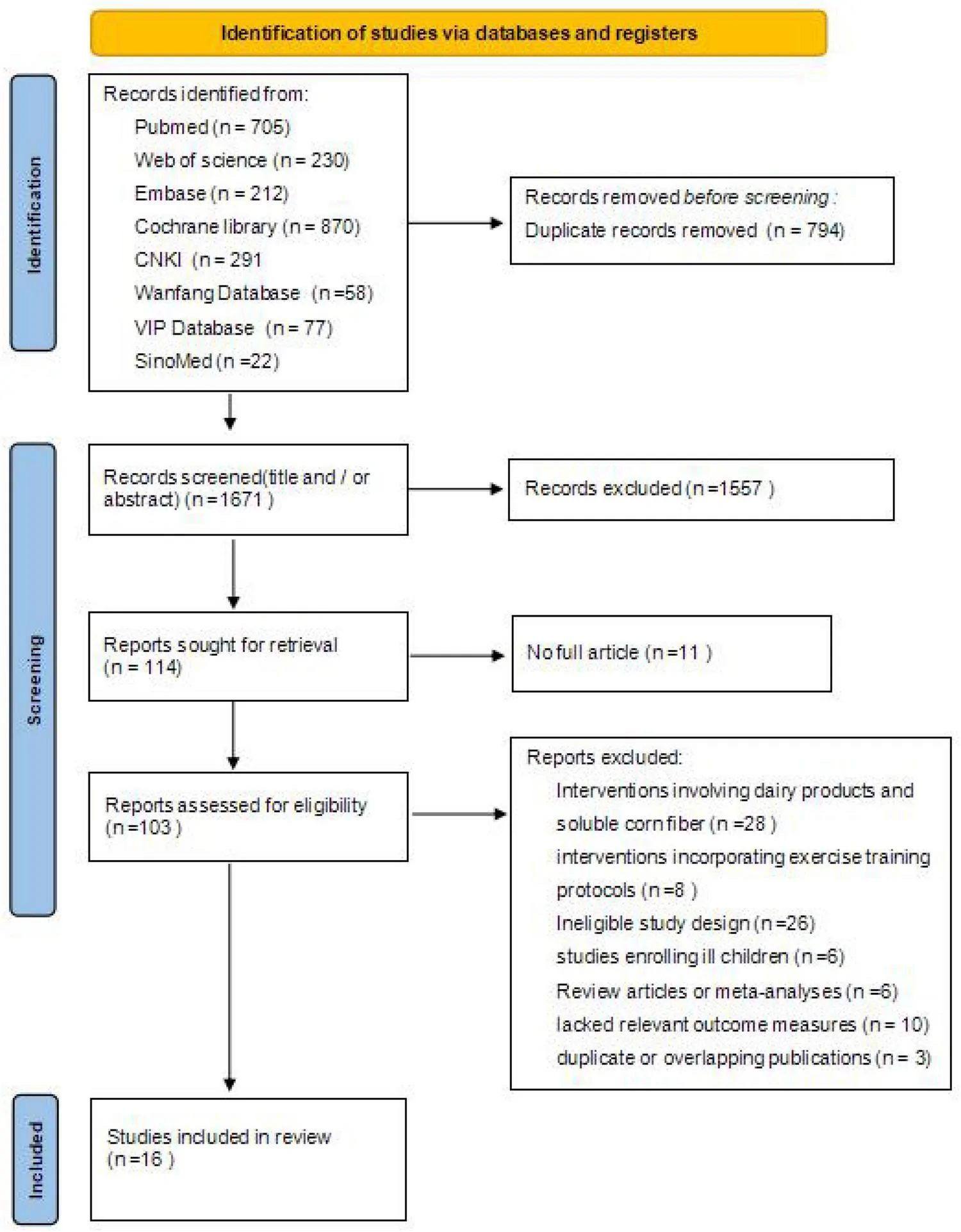
PRISMA flow diagram for record identification, screening, eligibility assessment, and study inclusion.

### 3.2 Risk of bias assessment

All 16 randomized controlled trials were independently evaluated by two investigators. Discrepant judgments were discussed, with a third investigator involved when consensus could not be reached.

Low risk of bias was assigned to 11 studies for the randomization process and to 14 studies for deviations from intended interventions. For missing outcome data, 10 studies were rated low risk, five raised some concerns, and one was rated high risk; identical counts were obtained for outcome measurement. Only two studies were considered low risk for selective reporting, whereas 14 raised some concerns because protocols or prespecified analyses were inadequately documented. Thus, at least some risk-of-bias concern was present in most trials (Figures 2, 3).

**FIGURE 2:**
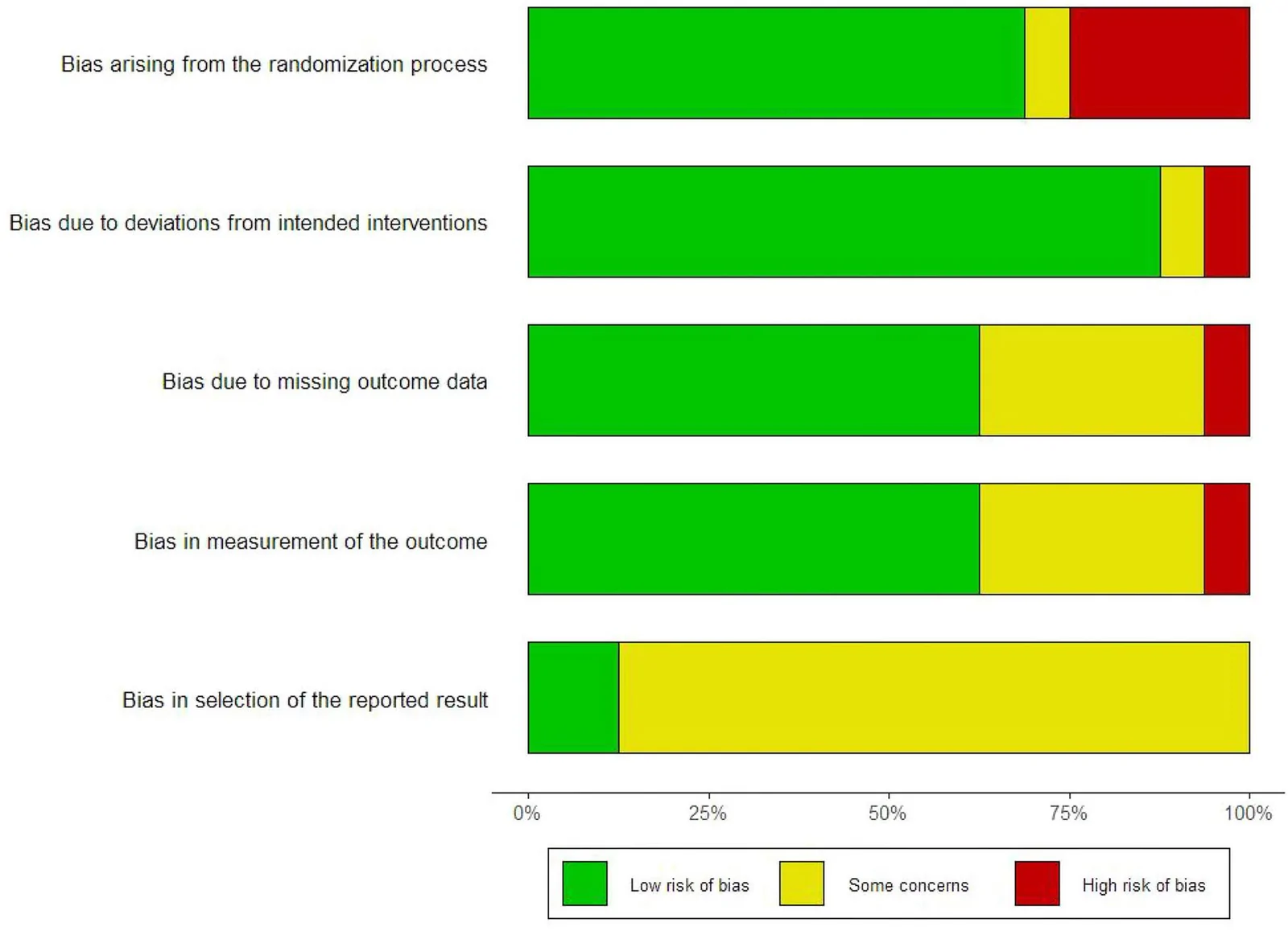
Distribution of RoB 2 judgments across risk-of-bias domains.

**FIGURE 3:**
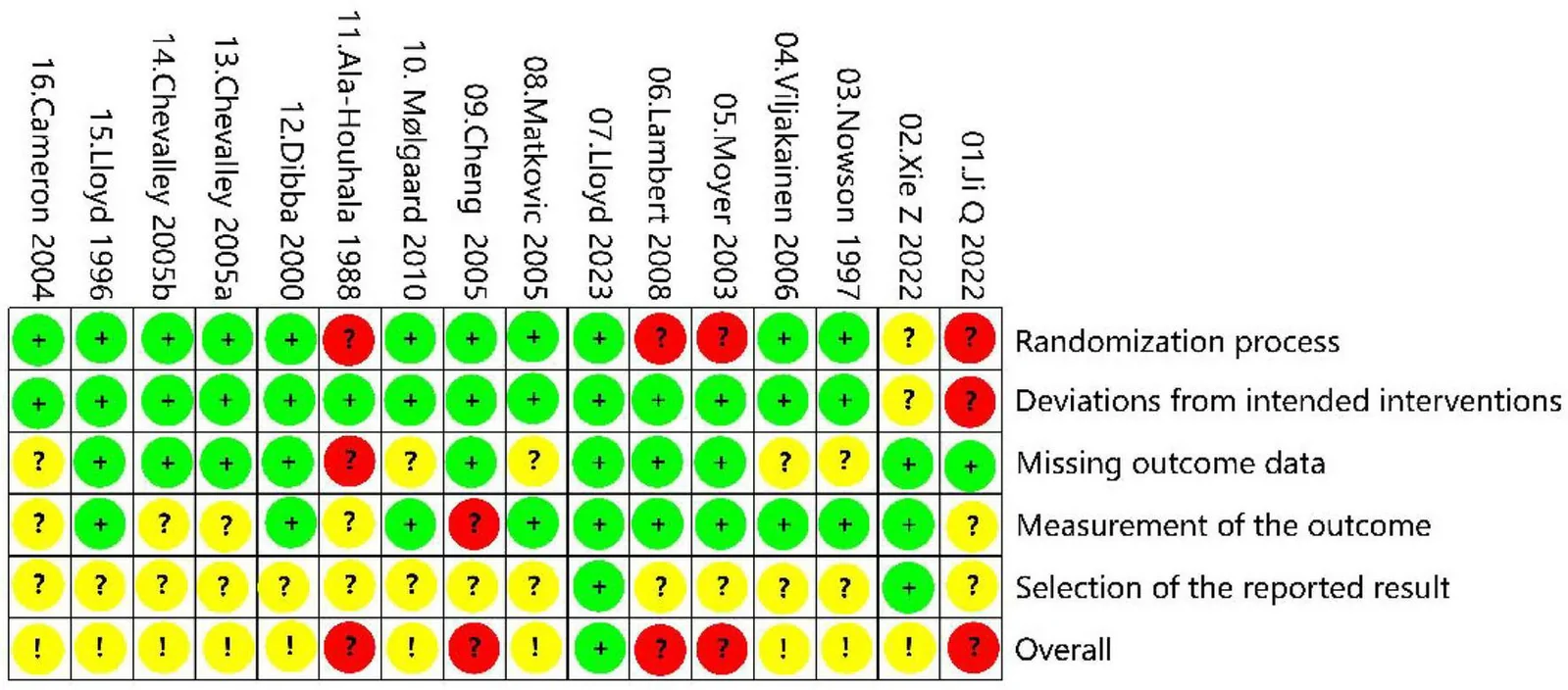
Summary of RoB 2 assessments.

### 3.3 Study characteristics

The 16 randomized controlled trials included approximately 2,100 children and adolescents and were conducted in China, the United States, Finland, Australia, Switzerland, the United Kingdom, Denmark, and The Gambia. The tested regimens were calcium alone, vitamin D alone, or calcium combined with vitamin D. Calcium doses ranged from 300 to 1,200 mg/d, and vitamin D doses ranged from 60 to 800 IU/d (1.5–20 μg/d). Intervention durations ranged from 6 to 48 months. Participants were 3–17 years old; most studies focused on pubertal children and predominantly enrolled girls (Table 1).

**TABLE 1:** Baseline characteristics of included studies.

References	Country	Sample size (T/C)	Sex (M/F)	Mean age (T/C)	Duration	Intervention	Dose	Outcome
Ala-Houhala et al. (30)	Finland	27/24	23/28	8–10/8–10	13 months	VitD	VitD_2_ 400 IU/d	➀➁
Cameron et al. (29)	Australia	51/51	0/102	10.3 ± 1.48/10.3 ± 1.48	24 months	Ca	Ca 1,200 mg/d	➀➁➂➃
Cheng et al. (28)	Finland	48/42	0/80	10–12/10–12	24 momths	Ca/VitD	Ca 1,000 mg/d+ VitD_3_ 5 μg/d	➀➁➂➃➄
Chevalley et al. (27)	Switzerland	77/67	0/144	8 ± 0.5/8 ± 0.5	12 months	Ca	Ca 850 mg/d	➀➁
Chevalley et al. (26)	Switzerland	116/116	232/0	7.42 ± 0.4/7.46 ± 0.39	12 months	Ca	Ca 850 mg/d	➀➁➂➃
Dibba et al. (25)	The Gambia	80/80	80/80	10.3 ± 1.0/10.3 ± 1.0	12 months	Ca	Ca 1,000 mg/d	➀➁
Ji et al. (32)	China	74/87	–	4.03 ± 0.77/4.28 ± 0.96	6 months	Ca+VitD	Ca 300 mg/d + VitD_3_ 60 IU/d	➀➁➂
Lambert et al. (24)	UK	45/44	0/89	12/12	18 months	Ca	Ca 792 mg/d	➁➄
Lloyd et al. (23)	USA	46/48	0/94	12 ± 0.5/11.8 ± 0.5	18 months	Ca	Ca 354 mg/d	➀➁➂➄
Lloyd et al. (22)	USA	44/44	0/88	13.9 ± 0.5/13.8 ± 0.6	24 months	Ca	Ca 500 mg/d	➀➁➂➄
Matkovic et al. (21)	USA	79/100	0/179	10.9 ± 0.9/10.8 ± 0.7	48 months	Ca	Ca 1,000 mg/d	➀➁➄
Mølgaard et al. (20)	Denmark	73/74	0/147	11.4 ± 0.2/11.4 ± 0.2	12 months	VitD	VitD_3_ 10 μg/d	➀➁➂➄
Moyer-Mileur et al. (19)	USA	35/36	0/71	11.9 ± 0.9/11.7 ± 0.8	12 months	Ca+VitD	Ca 800 mg/d + VitD_3_ 400 IU/d	➀➁
Nowson et al. (18)	Australia	42/42	0/84	14/14	12 months	Ca	Ca 1,000 mg/d	➀➁➂➃
Viljakainen et al. (17)	Finland	76/76	0/152	11.4 ± 0.4/11.4 ± 0.4	12 months	VitD	VitD_3_ 10 μg/d	➀➁➂
Xie (31)	China	50/50	47/53	8.22 ± 2.78/7.98 ± 2.02	12 months	VitD	VitD_3_ 800 IU/d	➀

Vit D, vitamin D; Ca, calcium; IU, international units; μg, microgram; mg, milligram; T, intervention group; C, control group; M, male; F, female. Outcomes: ➀ height; ➁ weight; ➂ lumbar spine BMD; ➃ femoral neck BMD; ➄ total body BMD.

### 3.4 Pairwise meta-analysis results

#### 3.4.1 Monthly height growth rate

Height outcomes were available from 14 of the 16 included studies. To account for differences in follow-up duration, monthly mean height growth was selected as the outcome metric. The intervention groups included 854 children and the control groups included 875. Between study heterogeneity was substantial (I^2^ = 85.6%), and the random-effects model showed no statistically significant difference between supplementation and control (MD = 0.0441 cm/month, 95% CI [−0.0114, 0.0996]; Figure 4).

**FIGURE 4:**
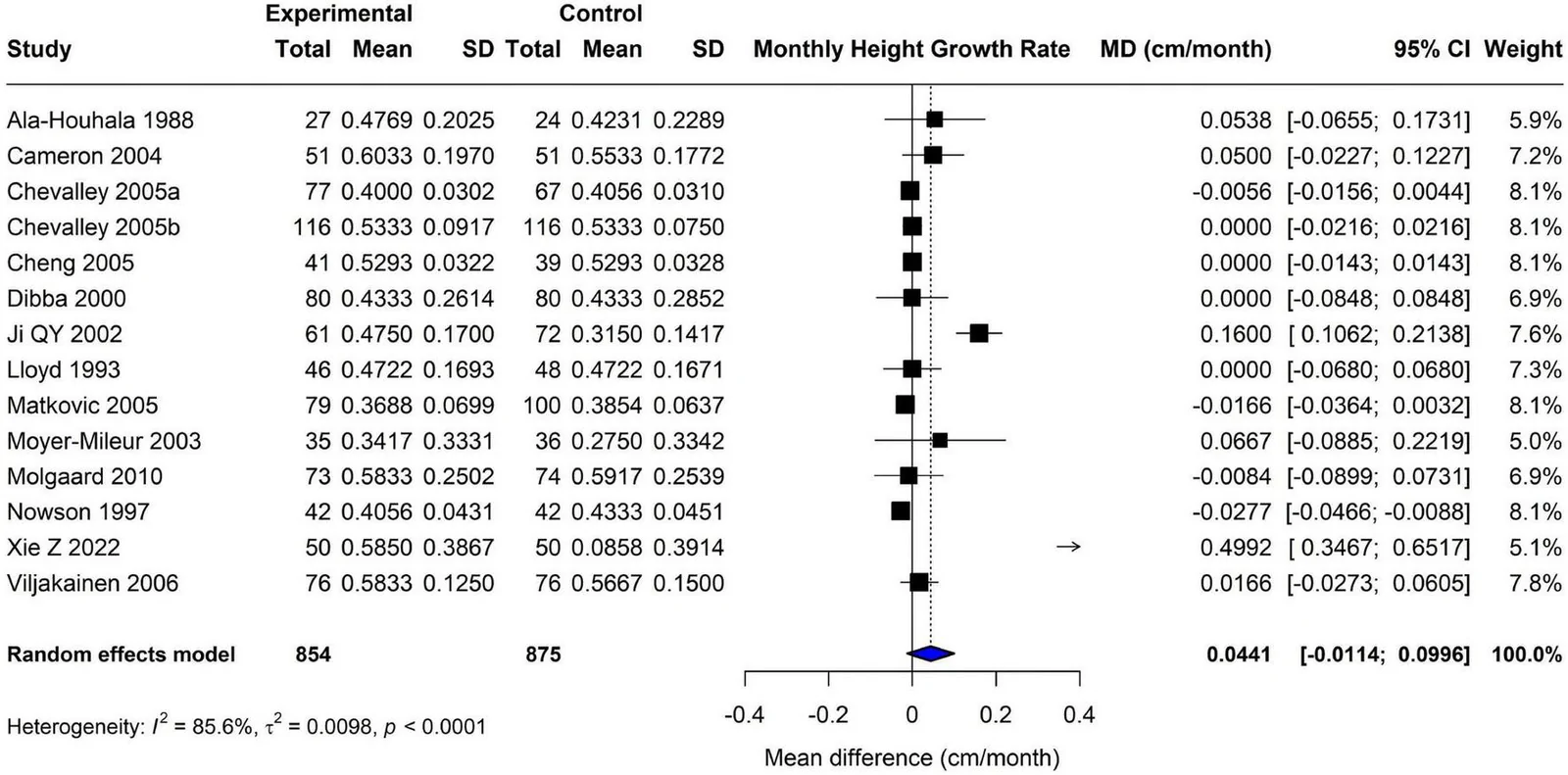
Relative effect forest plot of monthly height growth rate.

Leave-one-out sensitivity analysis showed that the pooled estimates remained positive or close to the null after individual studies were removed, with no material reversal in effect direction. Excluding Xie (31) attenuated the pooled estimate, indicating some influence on its magnitude, but did not change the overall interpretation (Supplementary Figure 11).

Subgroup analyses were performed by intervention duration and participant age. The between subgroup difference by duration was not statistically significant (*p* = 0.1519);The ≤12 month subgroup included nine studies (*N* = 1223; MD = 0.0680 cm/month, 95% CI [−0.0285, 0.1645]; I^2^ = 90.6%), whereas the >12 month subgroup included five studies (*N* = 506; MD = −0.0033 cm/month, 95% CI [−0.0178, 0.0111]; I^2^ = 18.1%) (Supplementary Figure 1). In contrast, differences among the three age groups were statistically significant (*p* < 0.0001). The estimated effects were 0.1600 cm/month (95% CI [0.1062, 0.2138]) in children aged 3–6 years (one study, *N* = 133), 0.0726 cm/month (95% CI [−0.0498, 0.1951]; I^2^ = 87.1%) in those aged 6–11 years (seven studies, *N* = 968), and −0.0064 cm/month (95% CI [−0.0257, 0.0129]; I^2^ = 33.7%) in those aged > 11 years (six studies, *N* = 628) (Supplementary Figure 6). Because the 3–6 year subgroup contained only one study and substantial heterogeneity persisted within the 6–11 year subgroup. Therefore, the observed subgroup difference should be considered exploratory rather than evidence of a definitive age-dependent treatment effect.

#### 3.4.2 Monthly weight growth rate

Body-weight data were reported in 12 studies, covering 1,483 children—743 in intervention groups and 740 in control groups. Weight change was expressed as kg/month to account for differences in follow-up duration. Between study heterogeneity was negligible (I^2^ = 0.0%); nevertheless, the random-effects model was retained because clinical heterogeneity was anticipated. Calcium and/or vitamin D supplementation did not significantly alter monthly weight gain relative to control (MD = 0.0075 kg/month, 95% CI [−0.0064, 0.0214]; Figure 5).

**FIGURE 5:**
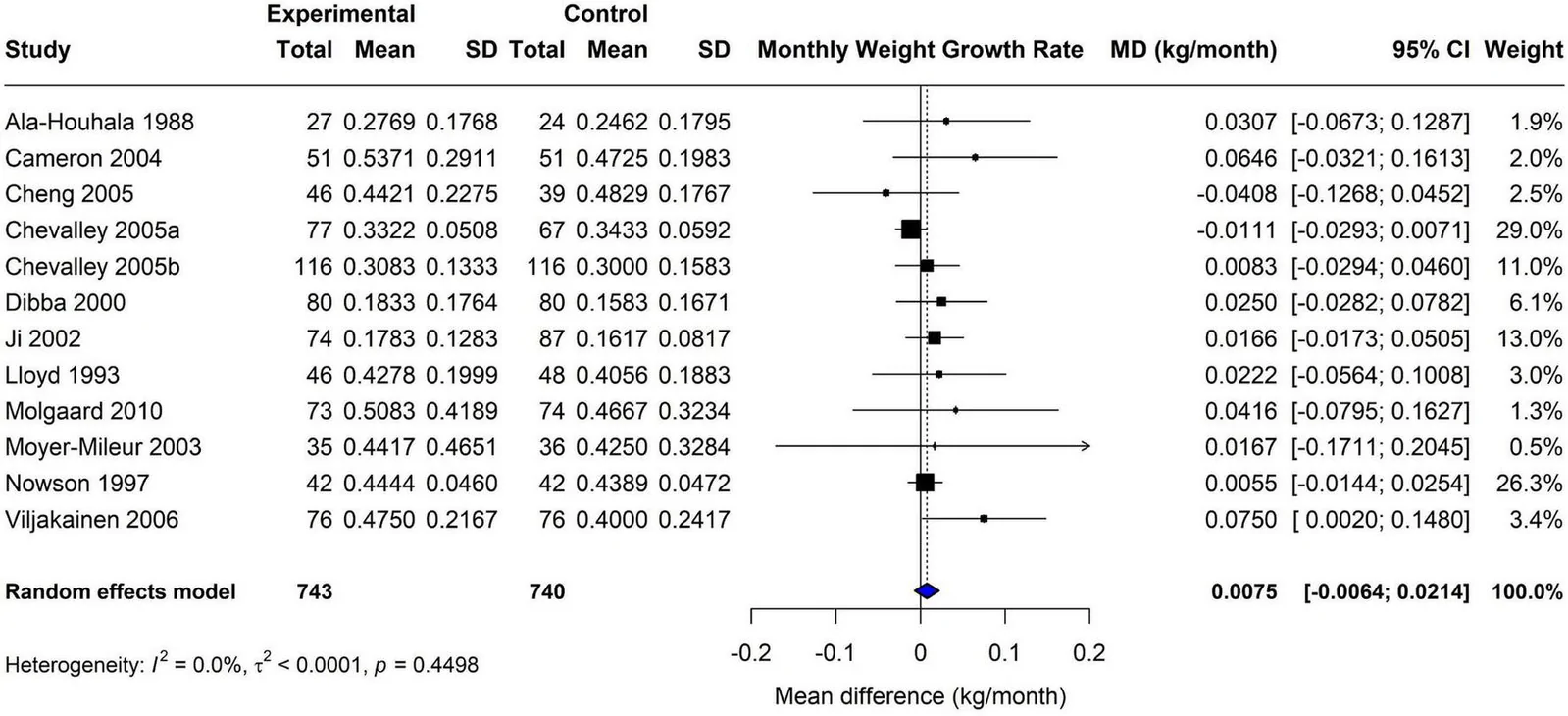
Relative effect forest plot of monthly Weight growth rate.

Exploratory subgroup analyses did not indicate meaningful variation in treatment effects by either age or intervention duration. When stratified by age, the estimated monthly weight gain was 0.0166 kg/month (95% CI [−0.0173, 0.0505]) among children aged 3–6 years (one study, *N* = 161), 0.0047 kg/month (95% CI [−0.0177, 0.0271]; I^2^ = 10.9%) among those aged 6–11 years (five studies, *N* = 689), and 0.0123 kg/month (95% CI [−0.0122, 0.0367]; I^2^ = 0%) among those aged > 11 years (six studies, *N* = 633). The between-subgroup difference was not statistically significant (*p* = 0.8221; Supplementary Figure 7).

A similar pattern was seen when studies were grouped by intervention duration. Eight studies with 1,151 participants contributed to the ≤12 month subgroup, for which the pooled estimate was 0.0066 kg/month (95% CI [−0.0080, 0.0212]; I^2^ = 11.1%), whereas the >12 month subgroup included four studies (*N* = 332; MD = 0.0161 kg/month, 95% CI [−0.0282, 0.0605]; I^2^ = 0%) (Supplementary Figure 2). Leave-one-out sensitivity analysis did not materially alter the direction or statistical interpretation of the pooled estimate (Supplementary Figure 12). Overall, the available pairwise evidence did not demonstrate a detectable effect of supplementation on monthly weight gain.

#### 3.4.3 Lumbar spine bone mineral density

Eleven studies reported changes in lumbar spine BMD, involving 1,383 children, including 692 in intervention groups and 691 in control groups. Heterogeneity was substantial (I^2^ = 94.1%). The random-effects pairwise analysis indicated a small positive effect of supplementation relative to control (MD = 0.0145 g/cm^2^, 95% CI [0.0040, 0.0249]; Figure 6).

**FIGURE 6:**
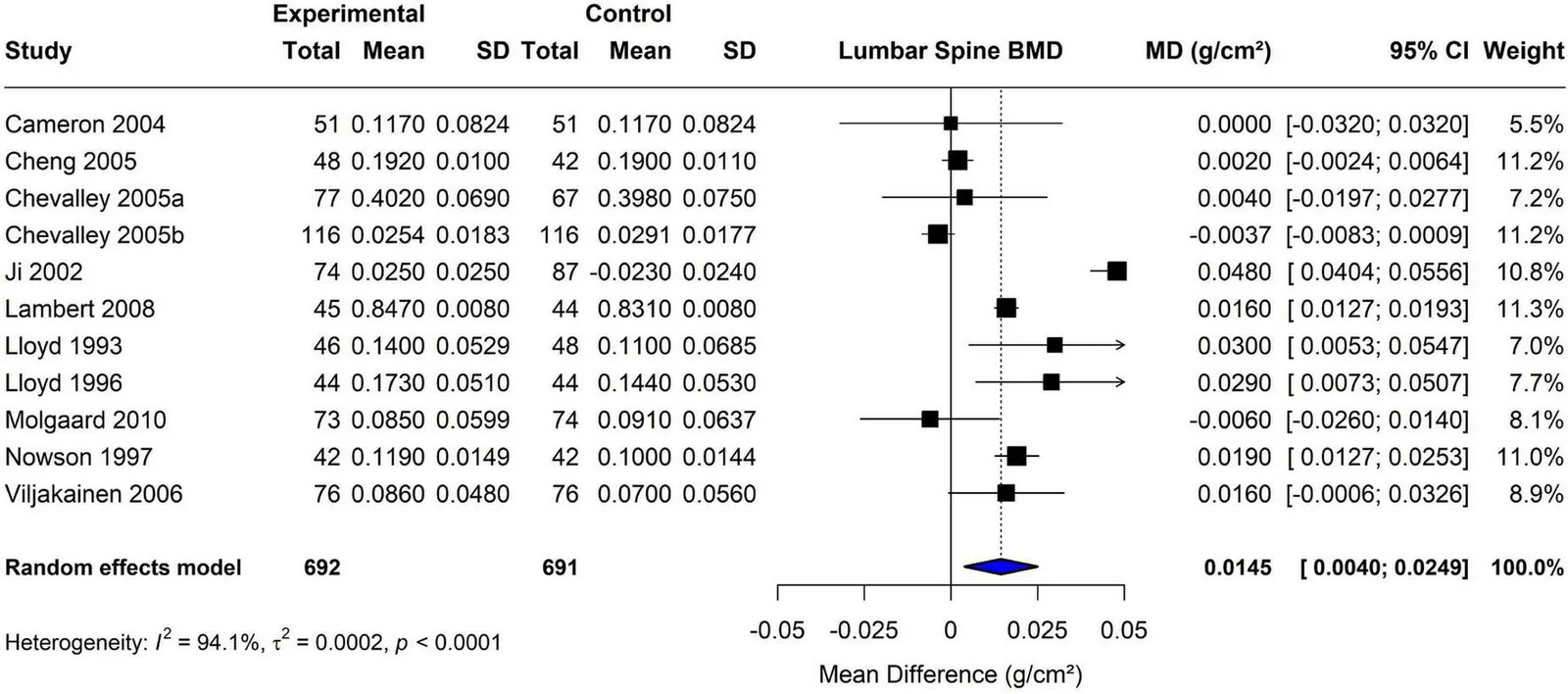
Relative effect forest plot of lumbar bone.

Exploratory age-stratified analysis showed a statistically significant between-subgroup difference (*p* < 0.0001). The estimated effect was 0.0480 g/cm^2^ (95% CI [0.0404, 0.0556]) among children aged 3–6 years (one study, *N* = 161), −0.0035 g/cm^2^ (95% CI [−0.0079, 0.0009]; I^2^ = 0%) among those aged 6–11 years (four studies, *N* = 625), and 0.0156 g/cm^2^ (95% CI [0.0074, 0.0237]; I^2^ = 85.7%) among those aged > 11 years (six studies, *N* = 597) (Supplementary Figure 8). However, the youngest subgroup was represented by only one study and substantial heterogeneity remained in the >11 year subgroup; therefore, the between-subgroup difference should be considered exploratory rather than evidence of a definitive age-dependent treatment effect.

No evidence of a difference according to intervention duration was observed (*p* = 0.9901). The ≤12 month subgroup included six studies (*N* = 920; MD = 0.0138 g/cm^2^, 95% CI [−0.0030, 0.0306]; I^2^ = 96.4%), whereas the >12 month subgroup included five studies (*N* = 463; MD = 0.0139 g/cm^2^, 95% CI [0.0026, 0.0252]; I^2^ = 86.8%) (Supplementary Figure 3). Substantial heterogeneity persisted within both duration subgroups. Leave-one-out analyses did not materially alter the direction of the pooled estimate, although the magnitude and precision varied modestly across exclusions (Supplementary Figure 13).

The pairwise evidence therefore suggested a small favorable effect on lumbar spine BMD, although the high heterogeneity limits the precision and generalizability of the pooled estimate. The observed age-subgroup differences should be considered exploratory and do not establish age as an independent source of heterogeneity.

#### 3.4.4 Femoral neck bone mineral density

Five studies reported femoral neck BMD outcomes, involving 652 children, including 334 in intervention groups and 318 in control groups. Between study heterogeneity was modest (I^2^ = 34.3%), and the random-effects pairwise analysis showed a small but statistically significant increase in femoral neck BMD with supplementation compared with control (MD = 0.0101 g/cm^2^, 95% CI [0.0064, 0.0138]; Figure 7).

**FIGURE 7:**
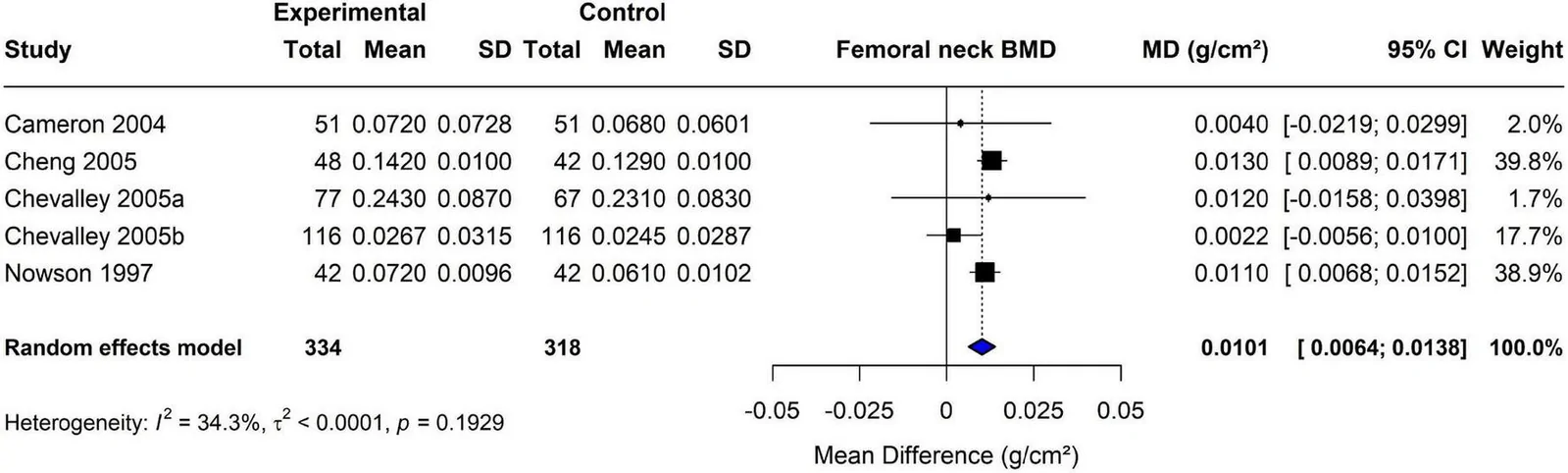
Relative effect forest plot of femoral neck mineral density.

Exploratory age-stratified analysis showed a statistically significant between-subgroup difference (*p* = 0.0226). Among children aged 6–11 years, three studies (*N* = 478) yielded an MD of 0.0030 g/cm^2^ (95% CI [−0.0042, 0.0102]; I^2^ = 0%), whereas among those aged > 11 years, two studies (*N* = 174) yielded an MD of 0.0120 g/cm^2^ (95% CI [0.0091, 0.0150]; I^2^ = 0%) (Supplementary Figure 9). However, given the small number of studies, particularly in the older subgroup, and the potential confounding of chronological age by pubertal stage, sex, body size, and baseline nutritional status, this finding should be considered exploratory rather than evidence of a definitive age-dependent treatment effect.

No statistically significant difference was observed according to intervention duration (*p* = 0.2326). The ≤12-month subgroup included three studies (*N* = 460; MD = 0.0077 g/cm^2^, 95% CI [0.0004, 0.0150]; I^2^ = 48.1%), whereas the >12 month subgroup included two studies (*N* = 192; MD = 0.0128 g/cm^2^, 95% CI [0.0087, 0.0169]; I^2^ = 0%) (Supplementary Figure 4). Leave-one-out analysis showed that the pooled point estimates remained positive after exclusion of individual studies, although their magnitude and precision varied (Supplementary Figure 14).

Overall, the pairwise analysis indicated a small positive difference in femoral neck areal BMD. However, the absolute effect was modest, and its clinical significance remains uncertain given variation in DXA measurement precision and the absence of a common clinically meaningful threshold across studies.

#### 3.4.5 Total body bone mineral density

Six studies reported total body BMD outcomes, involving 687 children, including 335 in intervention groups and 352 in control groups. Heterogeneity was substantial (I^2^ = 86.8%). The random-effects pairwise analysis showed a small positive effect relative to control (MD = 0.0110 g/cm^2^, 95% CI [0.0049, 0.0170]; Figure 8), but the lower confidence limit approached the null and the result should be interpreted cautiously.

**FIGURE 8:**
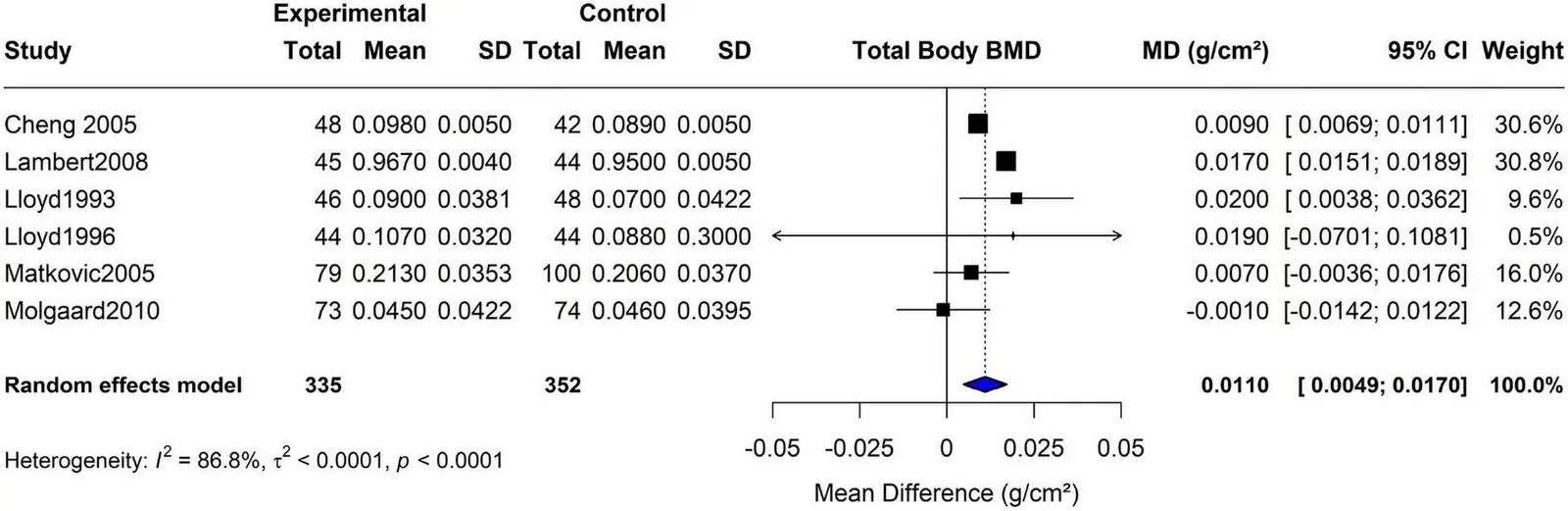
Relative effect forest plot of total body mineral density.

Exploratory age-stratified analysis showed no statistically significant between-subgroup difference (*p* = 0.4775). Among children aged 6–11 years, one study (*N* = 179) yielded an MD of 0.0070 g/cm^2^ (95% CI [−0.0036, 0.0176]), whereas among those aged > 11 years, five studies (*N* = 508) yielded an MD of 0.0117 g/cm^2^ (95% CI [0.0045, 0.0189]; I^2^ = 89.1%) (Supplementary Figure 10). Given that the younger subgroup was represented by only one study and substantial heterogeneity persisted in the older subgroup, these findings do not provide robust evidence of an age-dependent treatment effect.

The between-subgroup difference according to intervention duration was also not statistically significant (*p* = 0.0622). The ≤12 month subgroup included one study (*N* = 147; MD = −0.0010 g/cm^2^, 95% CI [−0.0142, 0.0122]), whereas the >12 month subgroup included five studies (*N* = 540; MD = 0.0127 g/cm^2^, 95% CI [0.0070, 0.0184]; I^2^ = 88.0%) (Supplementary Figure 5). Although the point estimates differed between duration subgroups, the subgroup test did not reach statistical significance, and the limited number of studies, particularly in the shorter-duration subgroup, precludes firm conclusions regarding a duration-dependent effect.

In leave-one-out analyses, the pooled point estimates remained positive, although the 95% CI crossed the null in some iterations, indicating some sensitivity of the precision and statistical significance to individual studies (Supplementary Figure 15). Overall, supplementation was associated with a small positive difference in total body areal BMD; however, substantial heterogeneity, limited subgroup evidence, and the small absolute magnitude of the effect warrant cautious interpretation, and its clinical significance remains uncertain. Exploratory meta-regression analyses examining the associations of age, intervention duration, and the proportion of female participants with monthly height growth rate, monthly weight growth rate, and lumbar spine BMD are presented in Supplementary Material 6.

### 3.5 Network meta-analysis

The network evidence plot (Figure 9) summarizes all treatment comparisons included in the network meta-analysis. Model convergence was assessed for all outcomes using trace plots and posterior density plots. The trace plots indicated stable chains with good mixing and no apparent systematic drift, whereas the posterior density plots were stable and predominantly unimodal. In the Brooks-Gelman-Rubin (BGR) diagnostics, both the median and the 97.5th percentile of the shrink factor rapidly approached 1 and remained close to 1 during subsequent iterations, further supporting adequate convergence of the Bayesian models (Supplementary Material 7). SUCRA values were calculated from the posterior distributions to quantify the relative ranking of the interventions. The feasibility of formal inconsistency assessment varied across outcome networks according to network topology. Node-splitting analyses were estimable for height, body weight, and lumbar spine BMD, and no statistically significant local inconsistency was detected. For total body BMD, node-splitting could not be estimated because the available multi-arm network structure did not permit independent separation of direct and indirect evidence. The femoral neck BMD network contained no evaluable closed loop, formal local inconsistency testing was not possible. Full network estimates are reported in Supplementary Material 8.

**FIGURE 9:**
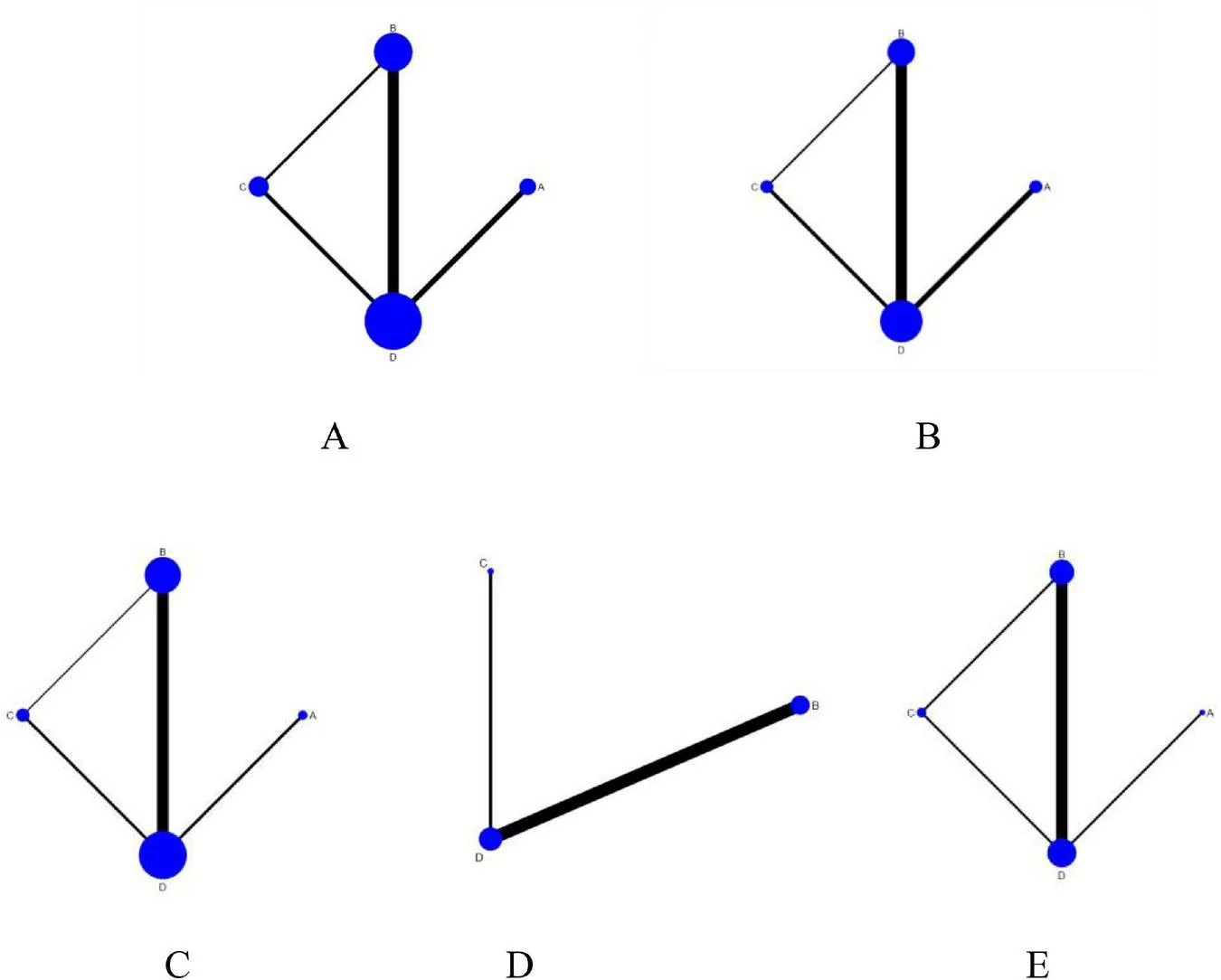
Evidence networks for the evaluated supplementation regimens. Panels show **(A)** height, **(B)** weight, **(C)** lumbar spine BMD, **(D)** femoral neck BMD, and **(E)** total body BMD. Edge width reflects the number of studies informing each comparison, whereas node area represents the total sample assigned to a regimen.

#### 3.5.1 Monthly height growth rate

Compared with placebo, the estimated effects on monthly height growth were vitamin D (MD = 0.111, 95% CrI [−0.006, 0.247]), calcium (MD = 0.006, 95% CrI [−0.075, 0.089]), and calcium plus vitamin D (MD = 0.053, 95% CrI [−0.079, 0.191]). All three CrIs included zero. Likewise, the CrIs for all pairwise comparisons among the active regimens included zero, providing no clear evidence that any supplementation strategy increased monthly height growth or that any active regimen was superior to another (Supplementary Table 1).

The ranking probabilities nevertheless placed vitamin D first, with a SUCRA value of 88.76%. Calcium plus vitamin D followed at 60.44%, whereas calcium and placebo yielded substantially lower values of 28.88% and 21.92%, respectively (Figure 10A). Vitamin D therefore carried the greatest posterior probability of occupying a favorable rank. That ranking should not, however, be read as proof of a treatment effect: uncertainty remained substantial, and the corresponding relative effect estimates did not provide definitive evidence either of benefit over placebo or of superiority over the other active regimens.

**FIGURE 10:**
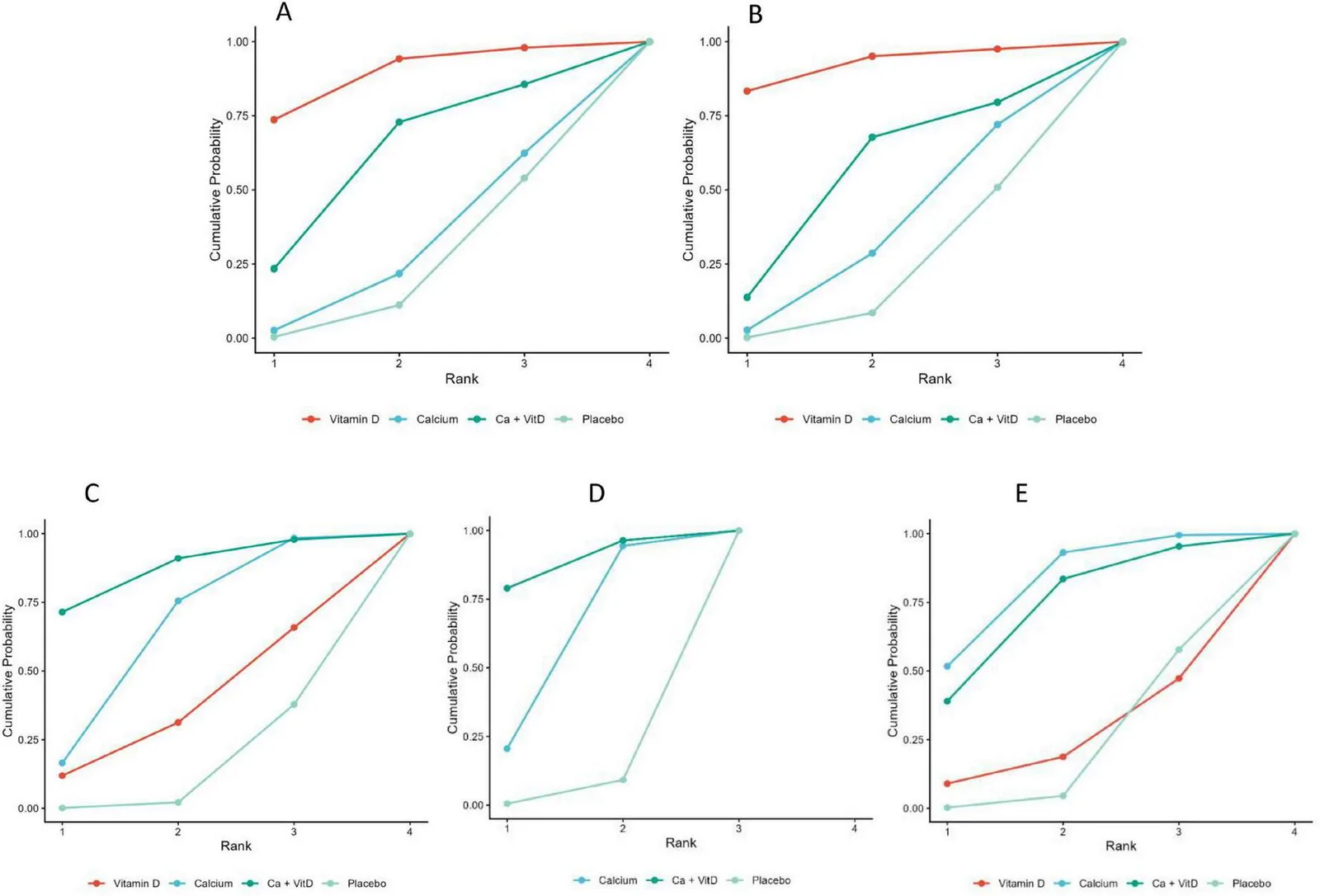
SUCRA curves for the principal outcomes: **(A)** height, **(B)** weight, **(C)** lumbar spine bone mineral density, **(D)** femoral neck bone mineral density, and **(E)** total body bone mineral density.

#### 3.5.2 Monthly weight growth rate

For monthly weight gain, the estimated effects relative to placebo were vitamin D (MD = 0.055, 95% CrI [−0.001, 0.112]), calcium (MD = 0.005, 95% CrI [−0.012, 0.032]), and calcium plus vitamin D (MD = 0.005, 95% CrI [−0.039, 0.043]). All CrIs included zero, and comparisons among the active regimens similarly showed no clear between treatment differences (Supplementary Table 2). These findings therefore provided no clear evidence that any supplementation strategy affected monthly weight gain.

Vitamin D ranked highest according to SUCRA (91.82%), followed by calcium plus vitamin D (54.52%), calcium (34.28%), and placebo (19.38%) (Figure 10B). Although vitamin D had the greatest probability of ranking first, its effect estimate remained compatible with the null, and the active treatment comparisons did not demonstrate a clear relative advantage. The SUCRA ranking should therefore be interpreted as probabilistic rather than as confirmatory evidence of treatment benefit.

#### 3.5.3 Lumbar spine bone mineral density

For lumbar spine BMD, the estimated effects relative to placebo were vitamin D (MD = 0.006, 95% CrI [−0.025, 0.035]), calcium (MD = 0.014, 95% CrI [0.0001, 0.029]), and calcium plus vitamin D (MD = 0.024, 95% CrI [−0.0010, 0.050]). The CrI for calcium narrowly excluded zero, suggesting a small positive effect relative to placebo, whereas the CrIs for vitamin D and calcium plus vitamin D included zero. Moreover, none of the comparisons among the active regimens clearly favored one treatment over another (Supplementary Table 3).

The SUCRA results yielded a somewhat different ordering. Calcium plus vitamin D ranked highest, with a value of 93.16%, followed by calcium at 51.58%, vitamin D at 37.28%, and placebo at 17.99% (Figure 10C). Of particular interest, the intervention assigned the highest ranking probability was not the intervention whose placebo comparison produced the only CrI excluding zero. This apparent divergence is readily explained by what SUCRA measures: it summarizes the posterior distribution of treatment ranks and does not represent either the statistical certainty or the absolute magnitude of an individual relative effect estimate. Accordingly, the high SUCRA value obtained for calcium plus vitamin D cannot be taken to show that the combined regimen was more effective than calcium alone.

#### 3.5.4 Femoral neck bone mineral density

In the network meta-analysis, the estimated effects relative to placebo were 0.008 g/cm^2^ for calcium (95% CrI [−0.0025, 0.017]) and 0.013 g/cm^2^ for calcium plus vitamin D (95% CrI [−0.002, 0.028]). Both CrIs included zero, providing no clear evidence that either supplementation strategy improved femoral neck BMD compared with placebo (Supplementary Table 4).

Calcium plus vitamin D had the highest SUCRA value (87.69%), followed by calcium (57.48%) and placebo (4.83%) (Figure 10D). However, this ranking should be interpreted cautiously. Although the combined regimen had the highest probability of occupying a favorable rank, its CrI versus placebo included zero, and the direct comparison with calcium was also compatible with no difference. Therefore, the network evidence did not establish superiority of calcium plus vitamin D over calcium or placebo for femoral neck BMD, and the SUCRA ranking should be regarded as exploratory given the sparse evidence base and imprecision of the estimates.

#### 3.5.5 Total body bone mineral density

For total body BMD, the estimated effects relative to placebo were vitamin D (MD = −0.001, 95% CrI [−0.024, 0.022]), calcium (MD = 0.012, 95% CrI [0.002, 0.023]), and calcium plus vitamin D (MD = 0.011, 95% CrI [−0.006, 0.029]). The estimate for vitamin D was close to the null, whereas calcium showed a small positive effect with a CrI excluding zero. The estimate for calcium plus vitamin D remained compatible with the null. Comparisons among the active regimens, however, did not clearly favor one supplementation strategy over another (Supplementary Table 5).

Calcium ranked highest according to SUCRA (81.57%), followed by calcium plus vitamin D (72.61%), vitamin D (24.98%), and placebo (20.84%) (Figure 10E). For this outcome, the SUCRA ranking was broadly concordant with the placebo-controlled estimates, as calcium ranked first and was the only active regimen with a CrI that excluded zero. Nevertheless, uncertainty in the comparisons among active treatments precluded a firm conclusion that calcium was superior to the other supplementation strategies.

### 3.6 Adverse reactions

Regarding safety, adverse events were reported only by Nowson and Lee; all consisted of mild gastrointestinal discomfort. No serious adverse events were reported. Although these findings suggest acceptable short-term tolerability of calcium and vitamin D related regimens, most included studies did not adequately report safety outcomes and lacked long-term follow-up. Consequently, no definitive conclusions regarding long-term safety can be drawn.

### 3.7 Publication bias

Comparison-adjusted funnel plots were examined for monthly height growth, monthly weight gain, and lumbar spine BMD (Supplementary Figures 16–18). Some asymmetry was observed for monthly height growth and lumbar spine BMD, whereas no marked asymmetry was apparent for monthly weight gain. However, the number of studies contributing to each outcome and individual treatment comparison was limited, reducing the reliability of visual assessment. Therefore, these plots were interpreted only as exploratory assessments of small-study effects, and neither symmetry nor asymmetry was considered sufficient evidence to confirm or exclude publication bias.

## 4 Discussion

### 4.1 Primary finding

This systematic review used conventional pairwise meta-analysis and Bayesian network meta-analysis (NMA) to evaluate the effects of calcium, vitamin D, and combined supplementation in healthy children and adolescents. Pairwise meta-analyses showed no statistically significant effects on monthly height growth or weight gain but identified small positive differences in areal BMD at the lumbar spine, femoral neck, and total body. Substantial heterogeneity was observed for lumbar spine and total body BMD, and the absolute effect sizes were small. The NMA similarly provided no clear evidence that any supplementation strategy improved monthly height growth or weight gain relative to placebo, nor did any active regimen show consistent superiority across outcomes. Treatment rankings varied by skeletal site, with calcium plus vitamin D ranking highest for lumbar spine and femoral neck BMD and calcium ranking highest for total body BMD. These SUCRA rankings, however, were not consistently supported by relative-effect estimates with credible intervals excluding zero and should therefore be regarded as exploratory rather than as evidence of treatment superiority. Overall, the findings indicate that calcium and/or vitamin D supplementation does not appreciably accelerate linear growth or weight gain in otherwise healthy children and adolescents. The detectable effects were confined primarily to small, site-specific differences in areal BMD, the clinical significance of which remains uncertain.

### 4.2 Comparison with previous research

The findings for linear growth are consistent with the current understanding of nutritional interventions in children. Height growth in children is not determined by any single micronutrient; rather, it is influenced by multiple factors, including genetic background, the growth hormone-IGF-1 axis, thyroid function, and the dietary environment (33–35). Once basic nutritional requirements have been met, additional calcium or vitamin D is unlikely to generate a sustained, clinically meaningful increase in stature. Randomized trials and previous syntheses have likewise reported little or no effect of supplementation on height, weight, or body composition in otherwise healthy children (36, 37). These findings do not negate the essential roles of calcium and vitamin D in normal growth and development. Instead, they underscore the distinction between correcting nutritional insufficiency and exceeding an individual’s normal growth potential.

Previous meta-analyses have found no clear, clinically important improvement in most pediatric BMD outcomes after vitamin D supplementation, with potential benefits largely confined to children with baseline vitamin D deficiency or to specific skeletal sites (15). Calcium supplementation has shown a similar pattern, with little change at the lumbar spine or femoral neck but occasional modest gains in total body or upper-limb BMD (38). The present comparison of calcium, vitamin D, and their combination reached much the same judgment: no strategy showed a consistent advantage across all skeletal sites, and the magnitude of benefit may vary with skeletal site, baseline nutritional status, maturational stage, and intervention duration. A meta-analysis of calcium supplementation and height reported an approximately 0.48 cm difference only among children whose calcium intake was inadequate at baseline and reached the recommended level after supplementation; the overall effect was not statistically significant (39). The evidence therefore favors correction of inadequate intake or biochemical deficiency rather than routine additional supplementation to promote height or broadly increase BMD in otherwise healthy children.

### 4.3 Potential mechanisms

#### 4.3.1 Differences between linear growth and bone mineral accrual

Linear elongation and bone mineralization proceed at the same time during childhood, but they are not the same biological process. Longitudinal growth occurs chiefly at the growth plate, where resting and proliferative chondrocytes divide, hypertrophy develops, extracellular matrix is formed, blood vessels invade, and endochondral ossification follows. The process is regulated by the growth hormone-IGF-1 axis, thyroid hormones, glucocorticoids, sex hormones, local paracrine signals, and mechanical loading (40–43).

Calcium and vitamin D act primarily through calcium-phosphorus homeostasis, intestinal calcium absorption, and mineralization of the bone matrix (6). In a child with marked calcium inadequacy or vitamin D deficiency, supplementation can remove a nutritional constraint on mineral deposition. Once supply is adequate, however, extra calcium or vitamin D cannot substitute for endocrine signaling or the genetic regulation of the growth plate.

Changes in body weight during childhood reflect the combined contributions of adipose tissue, lean mass, body fluids, organ tissue, and skeletal mass. Even when supplementation modestly increases bone mineral deposition, the contribution to total body weight is small and is generally insufficient to produce a clear between group difference in monthly weight gain. Randomized trials showing no significant effect of prolonged vitamin D supplementation on childhood weight or body composition are consistent with this physiology (36, 37). The near-zero effect on body weight and extremely low between study heterogeneity observed in the present study are therefore biologically plausible.

#### 4.3.2 Site-specific BMD responses and treatment rankings

Skeletal responses during growth may vary by anatomical site because regions differ in cortical and trabecular composition, bone turnover, mechanical loading, and geometry (44). The lumbar spine has a relatively high trabecular component, whereas the femoral neck contains both cortical and trabecular bone and is influenced by mechanical loading and local skeletal geometry. Total body BMD integrates measurements across skeletal regions with differing structural and metabolic characteristics. These biological differences may partly account for the variation in treatment estimates and SUCRA rankings across skeletal sites.

Nevertheless, site-specific treatment rankings should not be interpreted as evidence of comparative superiority. Although calcium plus vitamin D ranked highest for both lumbar spine and femoral neck BMD, the corresponding networks were sparse, and several relative-effect estimates were imprecise or compatible with no difference. A complementary biological role is plausible, because vitamin D facilitates calcium absorption whereas calcium provides the mineral substrate for bone mineralization. However, the present evidence does not establish a synergistic effect of combined supplementation or consistent superiority of any regimen across skeletal sites.

#### 4.3.3 Exploratory age-related differences

Exploratory age-stratified analyses showed statistically significant between-subgroup differences in monthly height growth, lumbar spine BMD, and femoral neck BMD, but not in monthly weight gain or total body BMD. These findings should not be interpreted as evidence of a causal or definitive age-dependent treatment effect. Several age strata were informed by only one or two studies, and substantial residual heterogeneity remained within some subgroups.

Chronological age is an imperfect proxy for biological and skeletal maturation. Both bone mineral accrual and linear growth change substantially during puberty, while peak calcium accretion occurs near, but does not necessarily coincide with, peak height velocity (45, 46). Age-defined subgroups may therefore differ simultaneously in pubertal stage, sex distribution, body size, hormonal status, physical activity, baseline calcium and vitamin D status, and supplementation regimen. These interrelated factors cannot be reliably separated using aggregate, study-level subgroup data. The observed age-related differences should therefore be considered hypothesis-generating and require confirmation in studies stratified by pubertal stage (preferably using Tanner staging) and baseline nutritional status.

### 4.4 Clinical implications

The principal clinical message is that meeting nutritional requirements should not be conflated with making a normally developing child grow taller. For children without inadequate intake, biochemical deficiency, skeletal disease, or another clinical indication, the present evidence does not support routine high-dose calcium or vitamin D supplementation solely to accelerate height or weight gain. Treatment rankings should not be used as stand-alone prescribing guidance because SUCRA values describe rank probabilities rather than the magnitude or certainty of benefit.

A targeted approach is more appropriate. Clinical assessment should consider dietary calcium intake, risk of vitamin D deficiency, sunlight exposure, growth and pubertal stage, relevant comorbidities, and risk of nutritional rickets. These factors should guide whether supplementation is required, which nutrient is most appropriate, and the dose and duration used. This interpretation is consistent with pediatric guidance that prioritizes adequate intake and prevention of deficiency rather than supplementation for height promotion (47–49).

Body size and adiposity may influence the biochemical response to vitamin D supplementation. Recent evidence indicates that the rise in serum 25(OH)D after supplementation varies with body weight and degree of adiposity (50), and studies in children and adolescents with overweight or obesity suggest that higher vitamin D doses may produce greater increases in circulating 25(OH)D concentrations (51, 52). However, these data do not establish a universally optimal or weight-adjusted dosing strategy. Because the present review lacked sufficient individual-level information on BMI, adiposity, baseline vitamin D status, and achieved 25(OH)D concentrations, we were unable to assess whether treatment effects differed according to body size or vitamin D dose requirements. Accordingly, our findings should not be used to define an optimal vitamin D dose for children with overweight or obesity, and future trials should evaluate dose-response relationships in relation to body size, adiposity, and baseline vitamin D status.

The clinical relevance of the observed BMD changes remains uncertain. The absolute effects were small, ranging from approximately 0.010–0.015 g/cm^2^ across skeletal sites, and should be interpreted in the context of DXA measurement precision, which varies by scanner, skeletal site, and calibration procedure. Because device- and site-specific least significant changes and clinically meaningful thresholds were not consistently available across the included trials, the clinical significance of these differences cannot be established. Moreover, small short-term changes in areal BMD do not necessarily translate into greater peak bone mass, improved bone geometry or material strength, or fewer fractures later in life (44, 53). Accordingly, these findings are best interpreted as modest improvements in site-specific areal BMD rather than evidence of clinically meaningful gains in skeletal strength or fracture prevention.

### 4.5 Strengths and limitations

This review has several strengths. First, it focused on otherwise healthy children and adolescents and separately evaluated conventional pairwise and Bayesian network evidence. Second, BMD outcomes were re-audited against the original reports and restricted to anatomically comparable DXA-derived areal BMD measurements, with lumbar spine, femoral neck, and total body outcomes analyzed separately. Third, network estimates, SUCRA rankings, convergence diagnostics, and inconsistency assessments were reported separately, allowing treatment rankings to be distinguished from effect estimates. The review also followed PRISMA and Cochrane methodological standards and used subgroup analyses, leave-one-out sensitivity analyses, meta-regression, and publication-bias assessments to examine result stability.

Despite the overall robustness of the findings, several limitations should be acknowledged. First, age- or sex-standardized growth z-scores and height-adjusted BMD were not consistently reported and therefore could not be synthesized. Monthly standardization accounts for differences in follow-up duration but does not remove differences in age, sex, pubertal stage, or body size. The pooled estimates should therefore be interpreted as average study-level randomized treatment effects rather than age- or body-size-standardized individual effects. Second, although the BMD synthesis was restricted to anatomically corresponding DXA-derived aBMD measurements expressed in g/cm^2^, differences in scanner systems, calibration procedures, skeletal acquisition protocols, and body- or bone-size adjustment could not be completely harmonized. These residual methodological differences may have contributed to the substantial heterogeneity observed for lumbar spine and total body BMD and limit the generalizability of the pooled raw MDs. Third, the age and duration subgroup analyses were based on aggregate data and several strata contained only one or two studies. Chronological age was also potentially confounded by pubertal stage, sex, body size, nutritional status, and intervention characteristics. Subgroup findings should therefore be regarded as exploratory. Relatively few studies contributed to femoral neck and total body BMD networks, reducing the precision of indirect comparisons and the stability of SUCRA rankings.

In addition, insufficient reporting of baseline nutritional status was another important limitation. Calcium and vitamin D interventions are most likely to benefit individuals with inadequate intake or biochemical deficiency, yet most studies did not report both dietary calcium intake and baseline serum 25(OH)D concentrations. Pooling nutritionally deficient and sufficient participants may therefore have diluted the true effects of supplementation. Information on BMI and adiposity was likewise insufficient to assess whether vitamin D dose requirements differed according to body size.

Future trials should stratify participants by baseline dietary calcium intake, serum 25(OH)D concentration, pubertal stage, and body size, and should report age- and sex-standardized growth measures and height-adjusted pediatric BMD where appropriate. Standardized DXA acquisition, calibration, and reporting procedures would improve cross-study comparability. Dose-response studies should specifically examine whether vitamin D requirements differ according to adiposity or body size rather than assuming that a single fixed dose produces equivalent exposure in all children. Longer follow-up is also needed to determine whether small short-term differences in areal BMD persist into peak bone mass and, importantly, whether they are associated with clinically relevant skeletal outcomes such as fracture risk.

## 5 Conclusion

In healthy children and adolescents, calcium and/or vitamin D supplementation did not meaningfully accelerate height or weight gain. The observed effects were small, related primarily to site-specific BMD, and did not establish consistent superiority of calcium, vitamin D, or their combination. These findings support targeted supplementation for inadequate intake, biochemical deficiency, or increased skeletal risk rather than routine use to promote growth. Well-designed trials stratified by baseline nutritional status and pubertal stage, with standardized outcomes and long-term follow-up, are needed to determine whether modest gains in childhood BMD improve peak bone mass, bone strength, or fracture risk later in life.

## Data Availability

The original contributions presented in this study are included in this article/Supplementary material, further inquiries can be directed to the corresponding authors.
