# Supplementary material for: Comparative effects of calcium, vitamin D, and combined supplementation on growth and bone mineral density in healthy children and adolescents: a systematic review and network meta-analysis
Source: Front Nutr. 2026 Oct 16;13:1979566. doi: 10.3389/fnut.2026.1979566 (PMC13719314; doi:10.3389/fnut.2026.1979566)
Supplement: Supplementary file 1 [file Table_1.docx]

Supplementary Material

# 1 S1:Search strategy

#1 "Calcium"[Mesh] OR "calcium"[Title/Abstract] OR "Calcium, Dietary"[Mesh] OR "Calcium, Dietary"[Title/Abstract] OR "Calcium supplementation"[Title/Abstract] OR "Vitamin D"[Mesh] OR "Vitamin D supplementation"[Title/Abstract] OR "calcium and vitamin D"[Title/Abstract]

#2 "Child"[Mesh] OR child*[Title/Abstract] OR "Adolescent"[Mesh] OR adolescen*[Title/Abstract] OR adolescen*,Female[Title/Abstract] OR adolescen*,male[Title/Abstract] OR teen*[Title/Abstract] OR youth*[Title/Abstract] OR "Pediatric"[Mesh] OR"pediatric"[Title/Abstract]OR schoolchild*[Title/Abstract]

#3 "Bone Density"[Mesh] OR bone mineral content*[Title/Abstract] OR bone mineral densit*[Title/Abstract] OR bone densit*[Title/Abstract] OR BMD[Title/Abstract] OR "bone age"[Title/Abstract] OR "body height"[Title/Abstract]OR "bone mass"[Title/Abstract] OR "bone loss"[Title/Abstract] OR "bone health"[Title/Abstract]

#4 #1 AND #2 AND #3

#5 randomized controlled trial[Publication Type] OR controlled clinical trial[Publication Type] OR random*[Title/Abstract] OR placebo[Title/Abstract] OR "clinical trial"[Title/Abstract] OR "RCT"[Title/Abstract]

#6 #4 AND #5

# 2 Supplementary Figures and Table

**S2: Meta-analysis of different intervention duration**

Figure S1.Meta-analysis of different intervention duration in monthly height growth rate

Figure S2.Meta-analysis of different intervention duration in monthly weight growth rate

Figure S3.Meta-analysis of different intervention duration in lumbar spine bone mineral density

Figure S4.Meta-analysis of different intervention duration in femoral neck bone mineral density

Figure S5.Meta-analysis of different intervention duration in total body bone mineral density

**S3: Meta-analysis of different age**

Figure S6.Meta-analysis of different age in monthly height growth rate

Figure S7.Meta-analysis of different age in monthly weight growth rate

Figure S8.Meta-analysis of different age in lumbar spine bone mineral density

Figure S9.Meta-analysis of different age in femoral neck bone mineral density

Figure S10. Meta-analysis of different age in total body bone mineral density

**S4: Sensitivity analysis**

Figure S11. Sensitivity analysis for monthly height growth rate

Figure S12. Sensitivity analysis for monthly weight growth rate

Figure S13. Sensitivity analysis for lumbar spine bone mineral density

Figure S14. Sensitivity analysis for femoral neck bone mineral density

Figure S15. Sensitivity analysis for total body bone mineral density

**S5: Comparison-adjusted funnel plots for assessment of small-study effects.**

Figure S16.Comparison-adjusted funnel plots for monthly height growth rate

Figure S17.Comparison-adjusted funnel plots for monthly weight growth rate

Figure S18. Comparison-adjusted funnel plots for lumbar spine bone mineral density

**S6: Meta regression**

| Factors | monthly height growth rate | | monthly weight growth rate | | lumbar spine bone mineral density | |
| --- | --- | --- | --- | --- | --- | --- |
|  | Coefficient | 95% CI | Coefficient | 95% CI | Coefficient | 95% CI |
| Age | -0.105 | (-0.210, -0.007) | 0 | (-0.037, 0.036) | -0.0020 | (-0.0271, 0.0231) |
| Duration | -0.007 | (-0.153,0.242) | 0.001 | (-0.043, 0.043) | -0.0099 | (-0.0332, 0.0158) |
| Female | -0.061 | (-0.168, 0.047) | ‑0.007 | (-0.039, 0.029) | 0.0035 | (-0.0233, 0.0320) |

**S7: MCMC convergence diagnostics**

Figure S19.Trace plots, posterior density plots, and BGR diagnostics of monthly height growth rate

Figure S20.Trace plots, posterior density plots, and BGR diagnostics of monthly weight growth rate

Figure S21.Trace plots, posterior density plots, and BGR diagnostics of lumbar spine bone mineral density

Figure S22.Trace plots, posterior density plots, and BGR diagnostics of femoral neck bone mineral density

Figure S23.Trace plots, posterior density plots, and BGR diagnostics of total body bone mineral density

**S8: Results of network Meta-Analysis**

Table S1. Results of network Meta-Analysis(Monthly Height Growth Rate)

|  | Vitamin D |  |  |  |
| --- | --- | --- | --- | --- |
| Calcium | 0.105 (-0.036, 0.266) | Calcium |  |  |
| Calcium+Vitamin D | 0.058 (-0.119, 0.251) | -0.047 (-0.196, 0.099) | Calcium+Vitamin D |  |
| placebo | 0.111 (-0.006, 0.247) | 0.006 (-0.075, 0.089) | 0.053 (-0.079, 0.191) | placebo |

Results are expressed as mean differences (MDs) with 95% credible intervals (CrIs). A CrI including zero indicates no statistically credible difference between treatments.

Table S2. Results of network Meta-Analysis(Monthly Weight Growth Rate)

|  | Vitamin D |  |  |  |
| --- | --- | --- | --- | --- |
| Calcium | 0.049 (-0.015, 0.109) | Calcium |  |  |
| Calcium+Vitamin D | 0.051(-0.017, 0.122) | 0.001 (-0.039, 0.054) | Calcium+Vitamin D |  |
| placebo | 0.055(-0.001, 0.112) | 0.005(-0.012, 0.032) | 0.005 (-0.039, 0.043) | placebo |

Results are expressed as mean differences (MDs) with 95% credible intervals (CrIs). A CrI including zero indicates no statistically credible difference between treatments.

Table S3. Results of network Meta-Analysis(lumbar spine bone mineral density)

|  | Vitamin D |  |  |  |
| --- | --- | --- | --- | --- |
| Calcium | -0.009 (-0.043, 0.024) | Calcium |  |  |
| Calcium+Vitamin D | -0.019 (-0.059, 0.020) | -0.01(-0.037, 0.017) | Calcium+Vitamin D |  |
| placebo | 0.006 (-0.025, 0.035) | 0.014 (0.0001, 0.029) | 0.024 (-0.001, 0.050) | placebo |

Results are expressed as mean differences (MDs) with 95% credible intervals (CrIs). A CrI including zero indicates no statistically credible difference between treatments.

Table S4. Results of network Meta-Analysis(femoral neck bone mineral density)

|  | Calcium |  |  |
| --- | --- | --- | --- |
| Calcium+Vitamin D | -0.005 (-0.024, 0.012) | Calcium+Vitamin D |  |
| placebo | 0.008 (-0.0025, 0.017) | 0.013 (-0.002, 0.028) | placebo |

Results are expressed as mean differences (MDs) with 95% credible intervals (CrIs). A CrI including zero indicates no statistically credible difference between treatments.

Table S5. Results of network Meta-Analysis(total body bone mineral density)

|  | Vitamin D |  |  |  |
| --- | --- | --- | --- | --- |
| Calcium | -0.014(-0.039, 0.012) | Calcium |  |  |
| Calcium+Vitamin D | -0.012(-0.042, 0.017) | 0.001 (-0.017, 0.019) | Calcium+Vitamin D |  |
| placebo | -0.001 (-0.024, 0.022) | 0.012 (0.002, 0.023) | 0.011 (-0.006, 0.029) | placebo |

Results are expressed as mean differences (MDs) with 95% credible intervals (CrIs). A CrI including zero indicates no statistically credible difference between treatments.
